# Identification of galectin-9 and its antibacterial function in Yellow River carp (*Cyprinus carpio haematopterus*)

**DOI:** 10.3389/fimmu.2025.1654890

**Published:** 2025-08-21

**Authors:** Li Wang, Yuanyuan Hu, Xudong Li, Ziyan Zhang, Nan Wang, Limin Chao, Chengfei Li, Pei Gao, Jinyou Ma, Lei Wang, Xiaojing Xia

**Affiliations:** ^1^ College of Animal Science and Veterinary Medicine, Henan Institute of Science and Technology, Xinxiang, China; ^2^ Postdoctoral Research Base, Henan Institute of Science and Technology, Xinxiang, China; ^3^ Postdoctoral Research Station in Biological Sciences, Henan Normal University, Xinxiang, China; ^4^ Fishery Technology Extension Station of Henan Province, Zhengzhou, China; ^5^ College of Life Science, Henan Normal University, Xinxiang, China; ^6^ Ministry of Education Key Laboratory for Animal Pathogens and Biosafety, Zhengzhou, China

**Keywords:** galectin-9, *Cyprinus carpio haematopterus*, innate immunity, agglutinating activity, binding ability

## Abstract

**Introduction:**

Galectin-9 is a β-galactoside-binding lectin that functions as a critical pattern recognition receptor (PRR) in the host immune system, initiating immune defense responses by recognizing and binding to pathogen-associated molecular patterns (PAMPs) on the surface of microorganisms. In this study, we identified and characterized a novel galectin-9 cDNA, designated CcGal-9, from Yellow River carp (*Cyprinus carpio haematopterus*).

**Methods:**

The full-length CcGal-9 cDNA was cloned and sequenced, and its structural features were analyzed. Tissue distribution of CcGal-9 mRNA was examined by quantitative real-time PCR. Expression changes following *Aeromonas hydrophila* and *Staphylococcus aureus* infections were evaluated. Recombinant CcGal-9 (rCcGal-9) was expressed in *Escherichia coli* BL21 (DE3), purified, and assessed for binding to various PAMPs and microorganisms. Agglutination assays and survival experiments were conducted to determine functional roles in immune defense.

**Results:**

The CcGal-9 cDNA is 963 bp in length and encodes a 320-amino acid protein with two distinct carbohydrate recognition domains (CRDs), characteristic of tandem-repeat type galectins. C*cGal-9* mRNA was predominantly expressed in the spleen, testicle, and head kidney, with lower levels in the liver and intestine. Upon bacterial infection, CcGal-9 expression was significantly upregulated in multiple immune-related tissues. Purified rCcGal-9 bound LPS, PGN, mannan, and both Gram-positive and Gram-negative bacteria, and exhibited broad-spectrum agglutination activity. Administration of rCcGal-9 markedly improved the survival rate of carp challenged with A. *hydrophila*.

**Discussion:**

These findings indicate that CcGal-9 is an important PRR in C. *carpio*, contributing to immune defense against pathogenic microorganisms through PAMP recognition and microbial agglutination. This study enhances our understanding of galectin-mediated immunity in teleost fish.

## 1 Introduction

Galectins are a family of β-galactoside-binding lectins characterized by the presence of a conserved carbohydrate recognition domain (CRD) that specifically binds to polysaccharides containing β-galactosides. This structural feature underpins the functional roles of these sugar-binding proteins in various biological processes (1, 2). Galectins are involved in a wide range of biological processes, including the regulation of embryonic development, tissue repair, adipogenesis, cancer progression, cell adhesion, apoptosis, inflammatory responses and the maintenance of immune homeostasis (3–6). The galectin protein family is defined by two key features: a high degree of amino acid sequence conservation and a strong affinity for β-galactosides. The mammalian galectins are classified into three structural subtypes: prototype galectins, which contain a single CRD; tandem-repeat galectins, which possess two distinct CRDs within a single polypeptide; and galectin-3, the sole chimeric galectin, which consists of a CRD linked to a non-lectin N-terminal domain (7).

Galectin-9 (Gal-9), also known as LGALS9, was first identified in rat embryonic kidney tissue in 1997 (8). As a member of the galectin family, Gal-9 is a soluble protein that is ubiquitously expressed across various tissues and exhibits a wide range of biological functions, particularly in immune regulation. It functions as a pattern recognition receptor (PRR) capable of recognizing pathogen-associated molecular patterns (PAMPs), and is involved in several critical immune processes, including opsonization, agglutination, phagocytosis, and microbial killing (9).Additionally, Gal-9 serves as an eosinophil chemoattractant by facilitating their recruitment via T cell activation, thereby enhancing immune responses. It plays a central role in modulating the Th17/Treg axis, contributing to both immunosuppression and T cell differentiation (10). Gal-9 exerts many of its immunomodulatory effects via binding to its ligand, T-cell immunoglobulin and mucin-domain containing molecule-3 (TIM-3), forming the Gal-9/TIM-3 signaling pathway (11). Moreover, Gal-9 has demonstrated tumor-suppressive properties through multiple mechanisms, including the induction of apoptosis, modulation of immune responses, and regulation of hematopoiesis (12–14). Beyond its roles in immunity and oncology, Gal-9 has also shown broad-spectrum antiviral activity against several clinically relevant viruses, including dengue virus, herpes simplex virus, hepatitis B virus, hepatitis C virus, HIV-1 and influenza virus (15).

Although substantial progress has been made in the study of galectin-9 in mammals, research on its function in fish remains limited. To date, galectin-9 have been identified and characterized in only a few teleost species. To date, Gal-9 has been reported in these fish species, such as *Trachidermus fasciatus* (16), *Micropterus salmoides* (17), *Boleophthalmus pectinirostris* (18), *Nibea albiflora* (19), *Planiliza haematocheilus* (20), *Oreochromis niloticus* (21) and so on. Previous studies have shown that galectin-9 is broadly distributed across various tissues in teleost fish. During bacterial or parasitic infections, galectin-9 functions as an acute-phase protein that responds rapidly, thereby playing a crucial role in host defense against microbial invasion (16–22). In teleosts, galectin-9 not only exhibits hemocoagulant activity but also directly binds bacterial glycans through its CRD, thereby facilitating the agglutination of both Gram-positive and Gram-negative bacteria. This interaction can result in disruption of bacterial cell walls and enhance macrophage-mediated phagocytosis, as well as promote the transcriptional upregulation of anti-inflammatory cytokines (16–22).

The Yellow River carp (*Cyprinus carpio haematopterus*) is one of the most widely farmed freshwater fish species in China, with considerable economic significance. However, the frequent occurrence of infectious diseases has led to a substantial reduction in its aquaculture productivity. As lower vertebrates, fish possess a relatively underdeveloped adaptive immune system; thus, advancing our understanding of their innate immune mechanisms is crucial for improving disease resistance. *A. hydrophila* is one of the most prevalent and virulent opportunistic bacterial pathogens in global freshwater aquaculture. It poses a substantial threat to aquaculture operations across regions including the Americas, Southeast Asia, and Africa. Characterized by rapid disease progression and high mortality rates, infections caused by *A. hydrophila* have resulted in considerable economic losses (23, 24). The molecular features of galectins, particularly galectin-9, remain largely unexplored in *C. carpio*. The aim of this study was to identify and characterize a galectin-9 homolog (*CcGal-9*) in *C. carpio*, and to investigate its molecular features, expression patterns, and immunological functions. We analyzed the gene sequence, protein structure, and tissue-specific expression of *CcGal-9* under normal and pathogenic conditions. We also evaluated the agglutination and binding activity of recombinant CcGal-9 (rCcGal-9) toward various bacterial strains, and assessed its *in vivo* protective effect against bacterial infection. These findings provide novel immunological insights and a scientific foundation for the development of effective disease management strategies in aquaculture.

## 2 Methods and materials

### 2.1 Fish and bacterial infection

Healthy *C. carpio* individuals (37 ± 2 g,17 ± 2 cm) were obtained from Tianhe Aquatic Products Co., Ltd., Yanjin, Xinxiang, Henan Province, China. Prior to injection, all fish were acclimated in freshwater in plastic aquaria (90 L; 67× 47×34 cm) at a controlled temperature of 20 ± 2°C, maintained using an automatic aquarium heater. The fish were fed commercial pellets twice daily. Individuals displaying signs of physical damage, abnormal behavior, or visible symptoms of disease were excluded from the study. For tissue distribution analysis, thirteen tissues were collected, including the kidney, head kidney, liver, skin, spleen, gill, heart, intestine, testis, ovary, swim bladder, brain, and muscle. Bacterial strains were inoculated at a 1:100 dilution into Luria-Bertani (LB) broth and incubated at 37°C with shaking at 180 rpm for 14-16 h until reaching the logarithmic growth phase. The cultures were then harvested by centrifugation and resuspended in sterile 0.65% NaCl to the desired concentration for subsequent experiments. In the *A. hydrophila* challenge group, each fish was injected with 200 μL of *A. hydrophila* in 0.65% NaCl (2×10^7^ CFU/mL) (25). In the *S. aureus* challenge group, each fish received 200 μL of *S. aureus* in 0.65% NaCl (4×10^7^ CFU/mL) (26). The control group was injected with an equivalent volume of 0.65% NaCl. Fish from both the control and challenge groups (5 per time point) were sampled at 3, 6, 12, 24, 48 and 72 hours post-injection (hpi). Tissues including the liver, spleen, kidney, head kidney, intestine and gill were collected. All samples were immediately flash-frozen in liquid nitrogen and stored at -80°C until RNA extraction.

### 2.2 Total RNA extraction and cDNA synthesis

Total RNA was extracted from the spleen of *C. carpio* using TRIzol reagent (TaKaRa, Japan, Cat# 9109) according to the manufacturer’s instructions. The RNA concentration and integrity were assessed as previously described (20, 26). First-strand cDNA was synthesized using the PrimeScript™ RT Reagent Kit with gDNA Eraser (TaKaRa, Japan, Cat# RR047A) according to the manufacturer’s protocol. The synthesized cDNA was stored at -20°C.

### 2.3 Gene cloning of *CcGal-9*

The open reading frame (ORF) of *CcGal-9* in *C. carpio* was identified by screening the NCBI database. Specific primers (*CcGal-9*-F and *CcGal-9*-R, as detailed in **Table 1**), which included *EcoR I* and *Xho I* (Cat# 1040S, 1094S) restriction sites, were designed to amplify the ORF according to the protocol for Ex Taq^®^ DNA Polymerase (Takara Biotech, Beijing, China, Cat# RR01A). PCR amplification was performed as follows: an initial denaturation step at 98°C for 5 min, followed by 30 cycles consisting of denaturation at 98°C for 10 s, annealing at 55°C for 30 s and extension at 72°C for 1 min, with a final extension step at 72°C for 3 min. The *CcGal-9* ORF was subsequently cloned into the pMD-19T vector (TaKaRa, Japan, Cat# 6013) and transformed into DH5α (Biomed, China, Cat# BC116-01). Positive clones were selected and sequenced by Sangon Biotech (Shanghai, China).

**Table 1:** Primers used in the present study.

Primer name	Sequence (5′-3′)	Purpose	Product size
*CcGal*-9-F	TCCGAATTCATGGCTTTTTATCAGCAACAA	ORF amplification	963bp
*CcGal*-9-R	GTGCTCGAGTTAAGCCTGCACTAAAGTC		
q*CcGal*-9-F	GGTTCCCAGCATACCCATCT	qRT-PCR analysis	300bp
q*CcGal*-9-R	AGGGATTGCAGGAGATGTTGAC		
*β-actin*-F	GAGTGATGGTTGGCATGGGA		120bp
*β-actin*-R	CCCAGTTGGTCACAATACCGT		

Enzyme restriction sites of *EcoR I* (GAATTC) and *Xho I* (CTCGAG) are underlined.

### 2.4 Bioinformatics analysis

Homology analysis was performed using the National Center for Biotechnology Information (NCBI) BLAST tool (http://blast.ncbi.nlm.nih.gov/Blast.cgi). The conserved domains were analyzed using SMART (http://smart.embl-heidelberg.de/smart/set_mode.cgi?NORMAL=1). The signal peptide was predicted using SignalP 4.1 (http://www.cbs.dtu.dk/services/SignalP/). The tertiary structure was predicted using Phyre2 (http://www.sbg.bio.ic.ac.uk/phyre2/html/page.cgi?id=index). Amino acid sequences of CcGal-9 were compared with those of other species using the Clustal X multiple alignment program. A phylogenetic tree was constructed based on the neighbor-joining method using MEGA 11.0, with 2000 bootstrap resampling.

### 2.5 *CcGal-9* expression by quantitative real-time PCR analysis

Quantitative real-time polymerase chain reaction (qRT-PCR) was employed to evaluate the expression levels of *CcGal-9* in healthy fish and to characterize its expression profiles following infection with *A. hydrophila* and *S. aureus*. QRT-PCR was conducted using an Applied Biosystems QuantStudio 5 Real-Time PCR System (Applied Biosystems, USA) with 2×Universal SYBR^®^ Green Fast qPCR Mix (ABclonal Biotechnology, China, Cat# RK21203), following the manufacturer’s protocols. The thermal cycling conditions were as follows: an initial denaturation at 95°C for 3 min, followed by 40 cycles of 95°C for 5 s and annealing at 60°C for 30 s. Specific primers (q*CcGal-9*-F and q*CcGal-9*-R, as listed in **Table 1**) were used to amplify the *CcGal-9* fragments, along with a reference gene, *β-actin* (*β-actin*-F and *β-actin*-R, also in **Table 1**). The relative expression of mRNA was quantified using the 2^-ΔΔCt^ method (27).

### 2.6 Expression and purification of rCcGal-9

Primers containing *EcoR I* and *Xho I* restriction sites, namely *CcGal-9*-F and *CcGal-9*-R (as listed in **Table 1**), were specifically designed to amplify the ORF of the *CcGal-9* gene. Recombinant plasmids pET-32a-*CcGal-9* and pET-32a were transformed into *E. coli* BL21 (DE3) competent cells following the protocol described in a previously published study (28). The transformed cells were subsequently inoculated at a 1:100 dilution into LB medium containing ampicillin and cultured at 37°C until the optical density at 600 nm (OD_600_) reached 0.6-0.8. Protein expression was induced by adding 0.5 mM isopropyl β-D-1-thiogalactopyranoside (IPTG). Following incubation at 37°C with shaking at 220 rpm for 6 h, the bacterial cells were harvested by centrifugation at 10,000 rpm and 4°C for 30 min to obtain the cell pellet. The pellet was washed three times with PBS, resuspended in PBS, and subjected to ultrasonic disruption on ice for 30 min. Subsequently, the lysate was centrifuged at 10,000 rpm and 4°C for 20 min to separate the supernatant from the cellular debris. SDS-PAGE analysis revealed that rCcGal-9 was expressed predominantly as inclusion bodies. The recombinant protein was then purified using the HyPur T Ni-NTA 6FF (His-Tag) PrePacked Gravity Column Kit (Sangon Biotech, Cat# C600332-0001) according to the manufacturer’s protocol. To facilitate protein refolding, dialysis was conducted at 4°C for 4–6 h using a stepwise urea gradient buffer containing 20 mM Tris-HCl, 300 mM NaCl, 10% glycerol, 1 mM reduced glutathione (GSH), 0.1 mM oxidized glutathione (GSSG), and sequentially decreasing concentrations of urea (6 M to 0 M). The dialyzed protein was subsequently concentrated by sucrose overlay at 4°C to obtain the desired final concentration. The concentration of the purified recombinant protein was quantified using the BCA assay. Western blot analysis was conducted to confirm the specificity of the 6×His-tag polyclonal antibody (Proteintech) against rCcGal-9. Additionally, the Trx-pET-32a recombinant protein (rTrx) was expressed and purified for use in subsequent experiments (28–30).

### 2.7 Bacterial agglutination assay

Gram-positive bacteria (*Micrococcus lysodeikticus*, *Bacillus subtilis*, *S. aureus* and *Streptococcus suis*) and Gram-negative bacteria (*A. hydrophila*, *Aeromonas veronii*, *Escherichia coli*, *Klebsiella pneumoniae*, *Pseudomonas aeruginosa*, *Vibrio fluvialis* and *Salmonella Pullorum*) were used for the bacterial agglutination assay. Briefly, bacteria cultured overnight were harvested and resuspended in TBS to a final concentration of 1×10^8^ CFU/mL. Subsequently, 20 µL of the bacterial suspension was mixed with 20 µL of rCcGal-9 (50 µg/mL) prepared in TBS. The mixture was incubated at 4°C for 2 h, after which agglutination was examined using oil immersion microscopy.

### 2.8 Binding analysis of rCcGal-9 with bacteria by Western blot

To evaluate the binding activity of rCcGal-9 to pathogenic bacteria identified in the preceding agglutination assay, Western blot analysis was performed according to the protocol described in a previous study (31). The specific procedure was as follows: bacteria cultured overnight were harvested, washed three times with TBS, and resuspended in TBS to an OD_600_ of approximately 1.0. Subsequently, 300 µL of the bacterial suspension (1×10^8^ CFU/mL) was mixed with 300 µL of rCcGal-9 (0.5 mg/mL) and incubated at 37°C with shaking at 180 rpm for 1 h. The bacteria were subsequently centrifuged to collect the pellet, which was resuspended and washed four times with TBS. Subsequently, 7% SDS was added, and the mixture was vortexed vigorously for 1 min to elute the proteins. Five times concentrated protein loading buffer (5×) was then added to the eluate, followed by heating the sample in a boiling water bath for 10 min. Protein samples were separated by 12% SDS-PAGE, followed by Western blot analysis using a 6×His tag antibody to assess the binding of rCcGal-9 to the bacteria. Purified rCcGal-9 served as a positive control in the assay.

### 2.9 Binding analysis of rCcGal-9 with bacteria and carbohydrates by ELISA

The binding of rCcGal-9 to *S. aureus* and *A. hydrophila* was assessed using the ELISA method to detect bacterial binding (29, 32). Briefly, 96-well plates were coated with 10 µg/mL of lipopolysaccharides (LPS), peptidoglycans (PGN) and mannan (Macklin Reagent) in a coating buffer consisting of 15 mM Na_2_CO_3_ and 35 mM NaHCO_3_. Plates were coated by adding 100 µL of solution per well and incubated at 4°C. Following removal of the coating solution, wells were blocked with PBS containing 0.25% BSA and 0.5% skim milk powder at 37°C for 2 h. After three washes, 20 µg of rCcGal-9, 20 µg of rTrx, or PBS were added to the wells and incubated at 25°C for 3 h. The primary antibody was a mouse anti-His antibody diluted 1:5000, and the secondary antibody was a horseradish peroxidase (HRP)-conjugated goat anti-mouse IgG antibody diluted 1:10,000 (SouthernBiotech). Following five washes, color development was initiated by adding a substrate solution containing 0.5 mg/mL tetramethylbenzidine (TMB) and 0.03% hydrogen peroxide (H_2_O_2_). The reaction was terminated by adding 2 M H_2_SO_4_, and the absorbance was measured at 450 nm using a microplate reader (Thermo Fisher Scientific).

In the polysaccharide inhibition assay, a fixed amount of rCcGal-9 protein (20 µg) was preincubated with various polysaccharides, including LPS, PGN and mannan, each at 10 mg/mL, for 3 h. The mixtures were subsequently incubated in microtiter wells coated with either *S. aureus* or *A. hydrophila* (100 µL, 1×10^7^ CFU/mL) for an additional 3 h. Antibody incubation, color development, and absorbance measurements were carried out as described above. Additionally, different concentrations of L-rhamnose, L-fucose, D-mannose, D-glucose, D-galactose, N-acetyl-D-mannosamine, D-xylose, sucrose and N-acetyl-D-glucosamine were preincubated with rCcGal-9 protein (20 µg) for 3 h, respectively. Subsequently, the mixtures were incubated in microtiter wells coated with *S. aureus* or *A. hydrophila* for an additional 3 h, followed by the procedures described above.

### 2.10 Effect of recombinant protein on survival rate of *C. carpio* infected with *A. hydrophila*

Healthy *C. carpio* individuals, averaging 20 g in weight, were randomly assigned to five groups, each containing 10 fish. A 0.65% NaCl solution was used for the dilution of rCcGal-9. Following purification, rCcGal-9 was diluted in physiological saline and administered intraperitoneally at graded doses of 1.00, 0.33, and 0.11 μg/g body weight per fish (33). Prior to injection, the bacterial suspension and recombinant protein were mixed in equal volumes (1:1). The negative control consisted of an equal-volume mixture of bacteria and 0.65% NaCl, while the positive control group received an injection of rCcGal-9 at a dose of 1 µg/g body weight. For intraperitoneal injection, five experimental groups were established: Group 1 received 200 µL of *A. hydrophila* bacterial suspension mixed with 0.65% NaCl; Group 2 received rCcGal-9 protein at a dose of 1 µg/g body weight; Group 3 received 200 µL of *A. hydrophila* suspension combined with 1 µg/g rCcGal-9 protein; Group 4 received 200 µL of *A. hydrophila* suspension mixed with 0.33 µg/g rCcGal-9 protein; and Group 5 received 200 µL of *A. hydrophila* suspension mixed with 0.11 µg/g rCcGal-9 protein. The activity status of *C. carpio* was monitored over a 72 hpi, and mortality rates were recorded.

### 2.11 Statistical analysis

All experiments were performed in triplicate. Statistical analyses were conducted using SPSS 17.0 software, with significance set at *P* < 0.05. Differences among groups were evaluated by one-way analysis of variance (ANOVA) followed by two-tailed Student’s t-tests. Figures were generated using GraphPad Prism 9.0.

## 3 Result

### 3.1 Cloning and sequence analysis of *CcGal-9*

**Figure 1:**
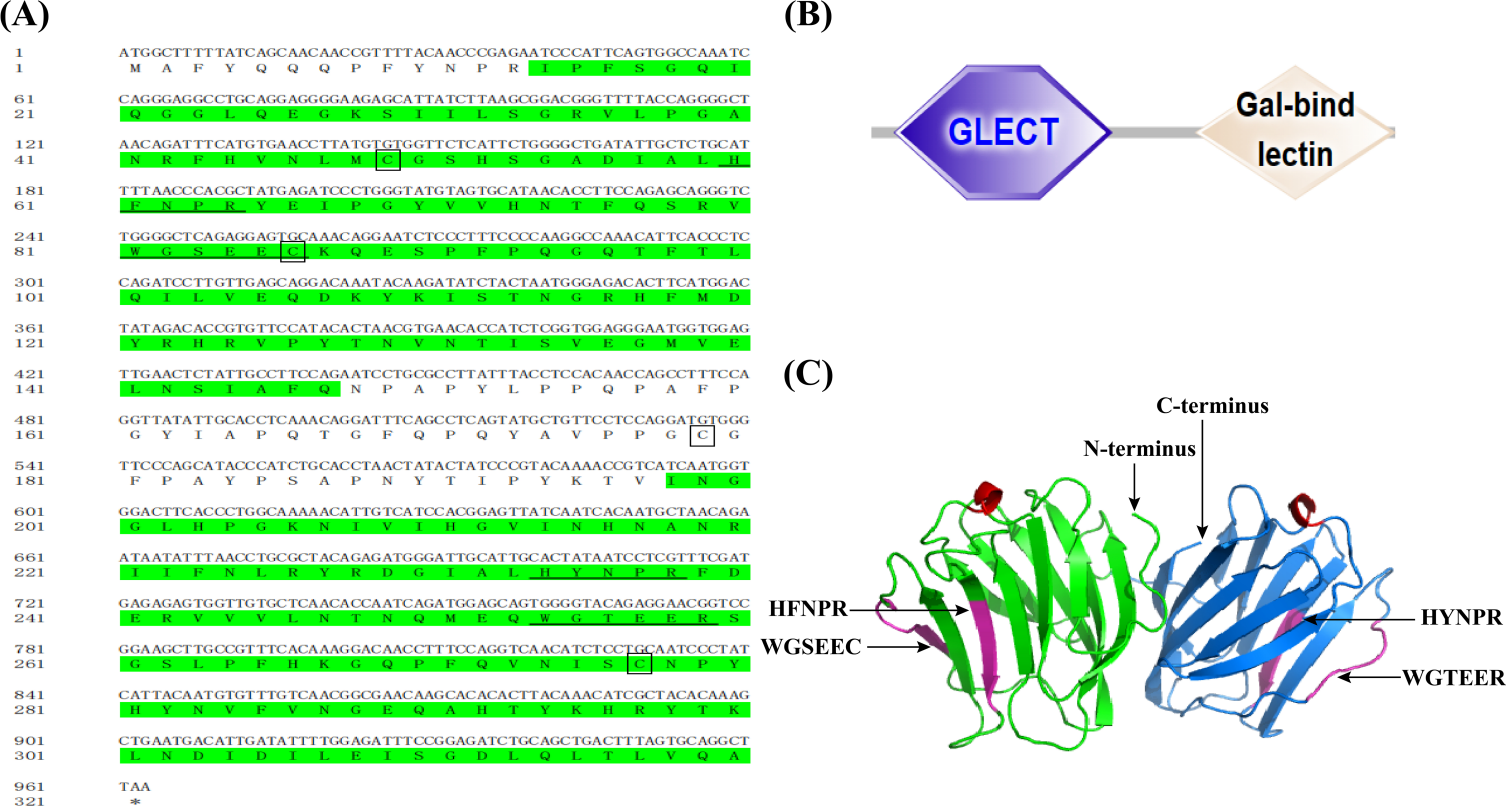
The sequences and predicted structure of *CcGal-9*. **(A)** The CRD are highlighted in green. Conserved motifs, including HFNPR, WGSEEC, HYNPR, and WGTEER, are underlined. The conserved cysteines are highlighted in boxes, and the stop codon is indicated by an asterisk (*). **(B)** Protein domains (N-CRD and C-CRD) of CcGal-9 predicted via SMART database.The hexagon and diamond represent CRDs. **(C)** CcGal-9 tertiary structure. Sugar binding motifs (HFNPR, WGSEEC HYNPR and WGTEER) are marked with arrows.

**Figure 2:**
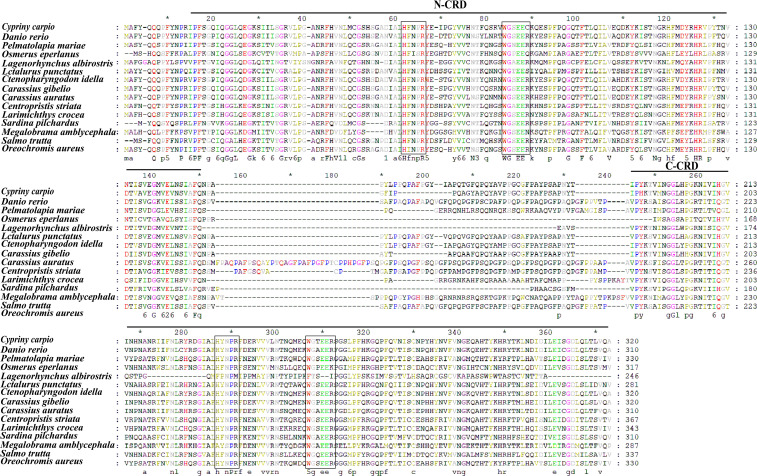
The multiple alignments. The boxes indicate the sugar-binding motifs (H-NPR and WG-EER) and the GenBank accession numbers of the amino acid sequences of the species are listed in **Table 2**.

**Figure 3:**
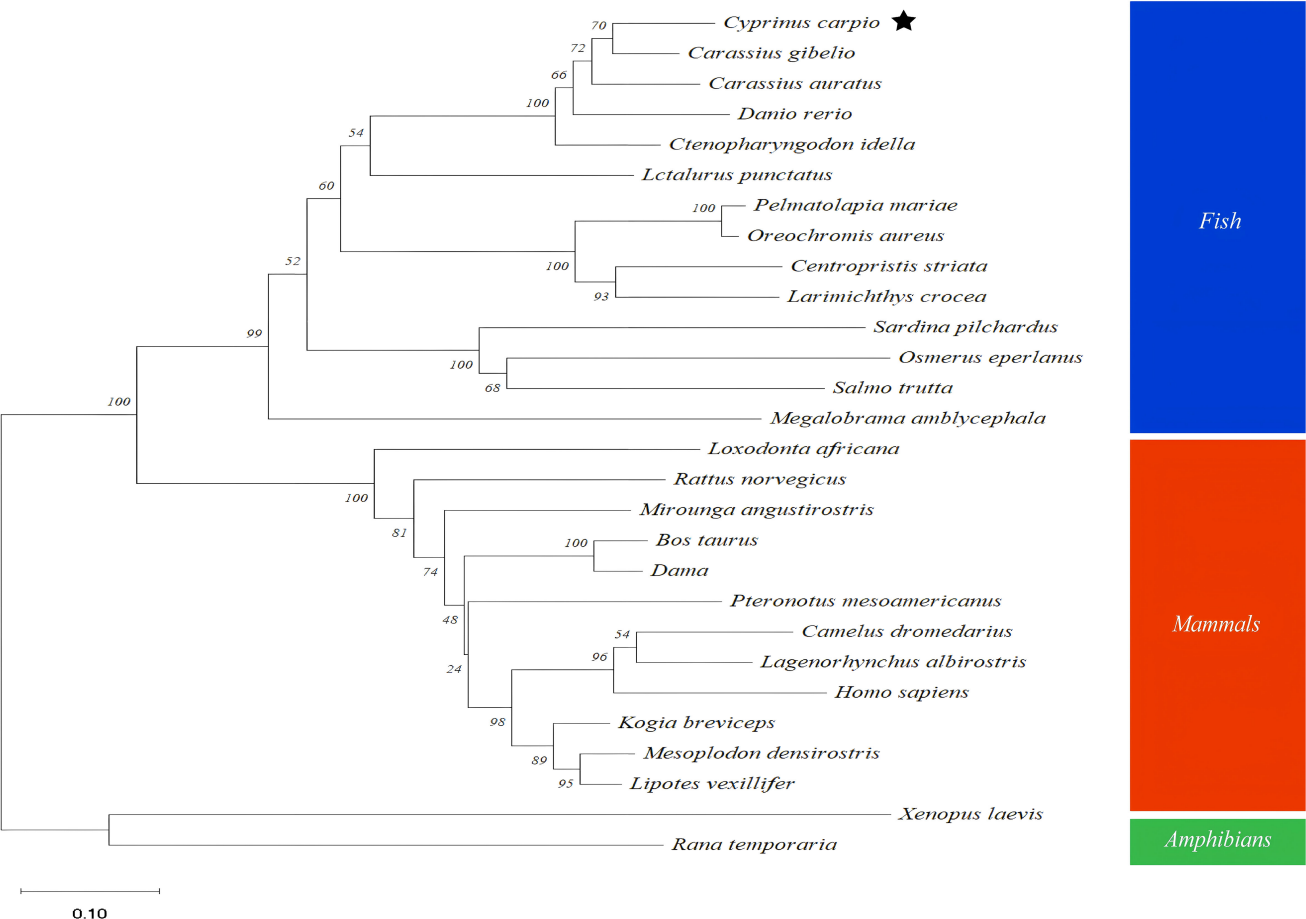
Phylogenetic tree analysis based on amino acids sequences from CcGal-9 and other Gal-9. The reliability of each node is estimated by bootstrapping with 2000 replications in MEGA 11.0. *Homo sapiens* (NP_001317092. 1), *Bos taurus* (NP_001015570. 1), *Dama* (XP_060998864. 1), *Mesoplodon densirostris* (XP_059936771. 1), *Kogia breviceps* (XP_058903876. 1), *Xenopus laevis* (XP_018109166. 1).

The full-length cDNA of *CcGal-9* identified in *C. carpio* is 963 bp in length and encodes a polypeptide of 320 amino acid residues, with a predicted molecular weight of 36.25 kDa and an isoelectric point of 8.26 (**Figure 1A**). No signal peptide or transmembrane domain was identified in the CcGal-9 protein. *In situ* motif analysis using the SMART program revealed that CcGal-9 contains two distinct CRDs, located at the N-terminus (residues 14-147) and C-terminus (residues 192-320), respectively (**Figure 1**). Each CRD contains distinct conserved motifs—HFNPR, WGSEEC, HYNPR and WGTEER—that are essential for β-galactoside binding. BLAST analysis showed that the CcGal-9 protein shares a high degree of sequence identity with galectin-9 proteins from *Carassius gibelio* (XP_052472718. 1, 88.44%), *Ctenopharyngodon idella* (XP_051717390. 1, 83.13%) and *Carassius auratus* (XP_026137648. 1, 82.81%) (**Figure 2** and **Table 2**). To investigate their phylogenetic relationships, a phylogenetic tree was constructed using MEGA 11.0, based on multiple sequence alignment results (**Figure 3**). Phylogenetic analysis revealed that CcGal-9 clusters closely with Gal-9 proteins from other teleost species.

**Table 2:** Biochemical properties of Gal-9 from *C. carpio* and other fish species.

Accession number	Species	Length (aa)	MW (KDa)	PI	Similarity (%)
XM_042739736.1	*Cyprinus carpio*	963	36.25	8.26	100
XP_052472718.1	*Carassius gibelio*	320	36.14	8.84	88.44
XP_051717390.1	*Ctenopharyngodon idella*	320	36.11	9.21	83.13
XP_026137648.1	*Carassius auratus*	310	35.07	7.79	82.81
NP_956366.1	*Danio rerio*	310	34.86	8.49	78.44
XP_054429155.1	*Pelmatolapia mariae*	355	39.38	9.48	56.36
XP_031602524.1	*Oreochromis aureus*	330	36.18	9.23	55.76
XP_017346983.1	*Lctalurus punctatus*	281	31.77	6.66	54.83
XP_010754381.2	*Larimichthys crocea*	343	37.49	9.1	53.35
XP_048063348.1	*Megalobrama amblycephala*	287	32.51	9.17	50.47
XP_059187938.1	*Centropristis striata*	367	40.13	9.54	50.14
XP_062393334.1	*Sardina pilchardus*	310	34.38	9.42	49.53
XP_029547510.1	*Salmo trutta*	337	38.04	9.82	48.97
XP_062342172.1	*Osmerus eperlanus*	317	35.06	8.89	47.35
XP_059990138.1	*Lagenorhynchus albirostris*	246	26.90	9.02	31.68

### 3.2 Expression of *CcGal-9* in different tissues

**Figure 4:**
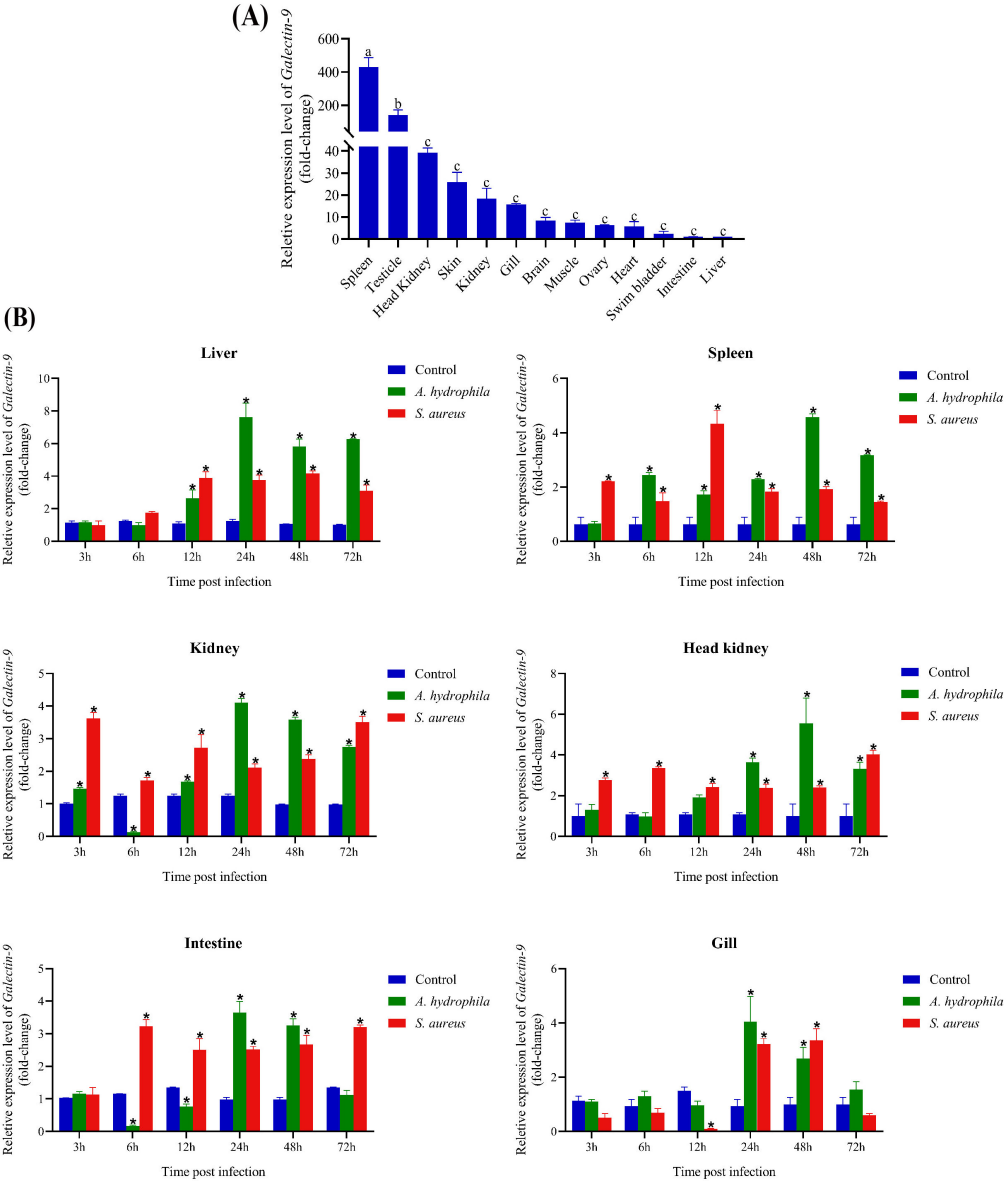
Expression profiles of *CcGal-9* transcripts. The mRNA expression level of *CcGal-9* relative to β-actin was analyzed using the 2^-ΔΔCt^ method. All data were expressed as mean ± SEM (n=5). n, the number of the experiment was performed. **(A)** Tissue distribution of *CcGal-9* in healthy *C. carpio*; a, b and c indicate the Duncan grouping in SPSS (*P* < 0.05). **(B)** Temporal expression patterns of *CcGal-9* in liver, spleen, kidney, head kidney, intestine and gill challenged with **(A)** *hydrophila* and *S. aureus*. The *CcGal-9* expression level in the control group at the same time point was chosen as calibrator (set as 1). Significant differences in the expression between the infected and control groups at the same time point were indicated with (*P < 0.05), Time points without significance markers indicate that the expression levels were not significantly different from those of the control group.

QRT-PCR was employed to analyze the mRNA expression profile of *CcGal-9* across 13 tissues of *C. carpio*, using *β-actin* as the internal reference gene. As shown in **Figure 4A**, *CcGal-9* mRNA transcripts were detected in all examined tissues, with the highest expression observed in the spleen, followed by moderate levels in the testicle, head kidney and kidney. Relatively high expression levels were also observed in the brain, muscle, ovary, swim bladder and intestine, whereas the lowest expression was detected in the liver.

### 3.3 Temporal expression patterns of *CcGal-9* after *A. hydrophila* and *S. aureus* infection

To explore the role of *CcGal-9* in the immune response to bacterial infections, qRT-PCR was conducted to evaluate its expression in key immune-related tissues—including the spleen, head kidney and kidney—as well as in gill tissues, following infection with *A. hydrophila* and *S. aureus* (**Figure 4B**). Following exposure to *A. hydrophila*, *CcGal-9* mRNA expression was significantly upregulated in the liver, spleen, head kidney and gill tissues. In the liver, *CcGal-9* transcript levels progressively increased from 12 to 72 hpi, with a peak observed at 24 hpi compared to the control group. In the spleen, a continuous increase in *CcGal-9* transcript levels was observed from 6 to 72 hpi, reaching a peak at 48 hpi. In the kidney, *CcGal-9* expression was significantly upregulated at 24, 48 and 72 hpi, while a notable downregulation was detected at 6 hpi. In the gill tissue, a significant upregulation of *CcGal-9* expression was observed between 24 and 48 hpi, with the peak expression occurring at 24 hpi. Conversely, *CcGal-9* mRNA expression was downregulated in both kidney and intestinal tissues (*P* < 0.05). *In vivo* studies in the kidney showed a significant upregulation of *CcGal-9* transcripts at 3, 12, 24, 48 and 72 hpi, with a notable downregulation observed at 6 hpi. Similarly, no significant changes in *CcGal-9* expression were observed in the intestine during the first 3 hpi. At 6 and 12 hpi, *CcGal-9* expression was downregulated, whereas it was significantly upregulated at 24 and 48 hpi, with the peak expression occurring at 24 hpi.

As shown in **Figure 4B**, following the challenge with *S. aureus*, *CcGal-9* mRNA expression was significantly increased in the liver, spleen, kidney, head kidney and intestinal tissues, reaching peak levels at different time points. Notably, *CcGal-9* mRNA expression was downregulated in the intestinal tract. In the gill, *CcGal-9* expression remained at baseline levels at 3, 6 and 72 hpi, while a significant downregulation was observed at 12 hpi. In contrast, *CcGal-9* expression was significantly upregulated at 24 and 48 hpi, with the highest expression recorded at 48 hpi.

### 3.4 Recombinant CcGal-9 expression, purification and Western blot analysis

**Figure 5:**
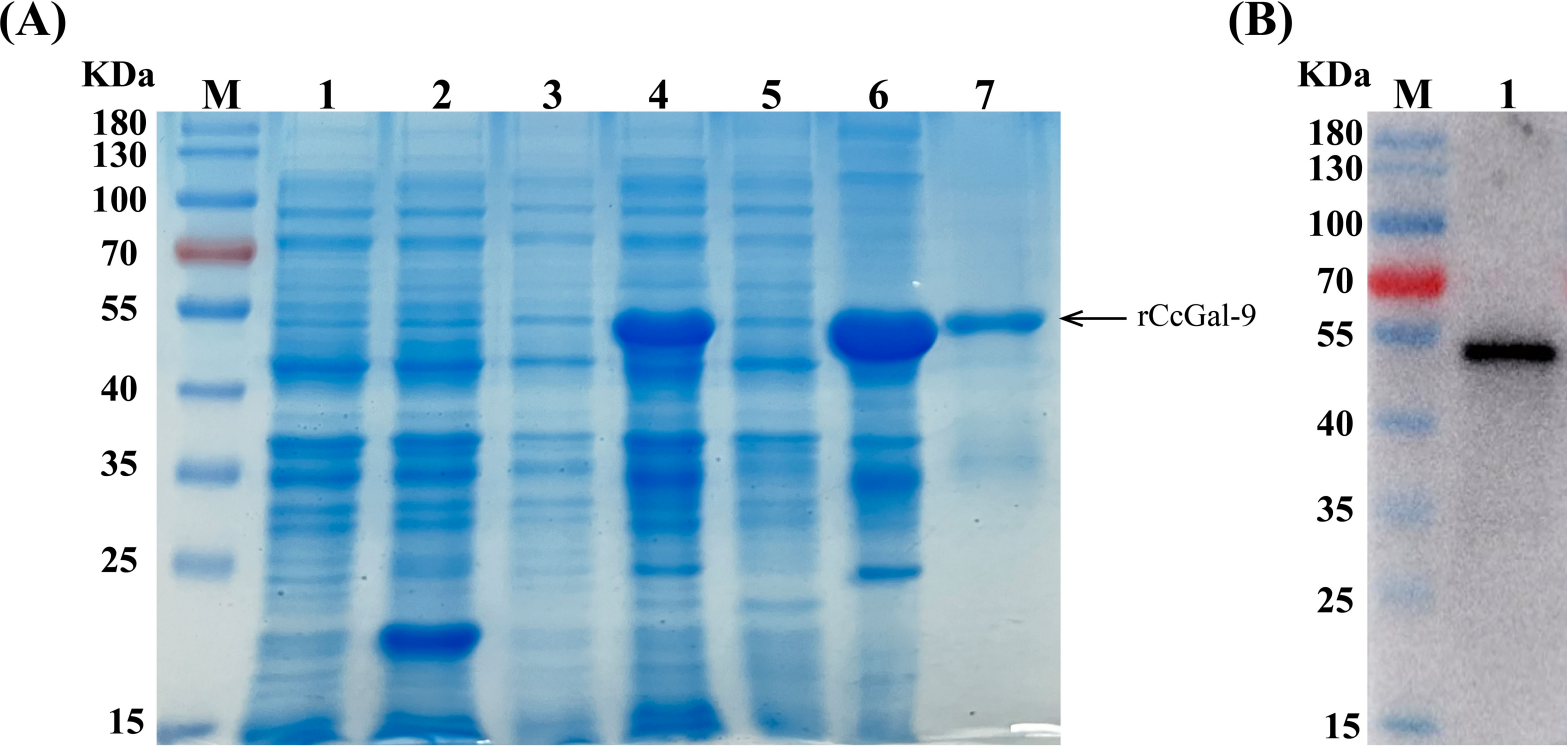
Expression and purification of rCcGal-9. **(A)** SDS-PAGE profile of rCcGal-9. Lane M, protein marker; Lane 1, pET-32a (non-induced); Lane 2, pET-32a (induced); Lane 3, pET-32a-*CcGal-9* (non-induced); Lane 4, pET-32a-*CcGal-9* (induced); Lane 5, pET-32a-*CcGal-9* (supernatant protein induced); Lane 6, pET-32a-*CcGal-9* (inclusion body protein induced); Lane 7, the purified rCcGal-9. **(B)** Western blot analysis of antibody against rCcGal-9. Lane M, protein marker; Lane 1, target band: anti-6 × His-tag antibody to bind rCcGal-9 in Western blot analysis.

To investigate the biological function of CcGal-9, this study successfully expressed recombinant CcGal-9 (rCcGal-9) using a prokaryotic expression system. As shown in **Figure 5**, the plasmid pET-32a-*CcGal-9* was transformed into *E. coli* BL21 and subsequently purified using a Ni-NTA gravity column. Recombinant CcGal-9 was obtained in the form of inclusion bodies. Protein analysis via 12% SDS-PAGE revealed a prominent band corresponding to an approximate molecular weight of 54 kDa (**Figure 5A**, lane 7).

### 3.5 Bacterial agglutination activity of rCcGal-9

**Figure 6:**
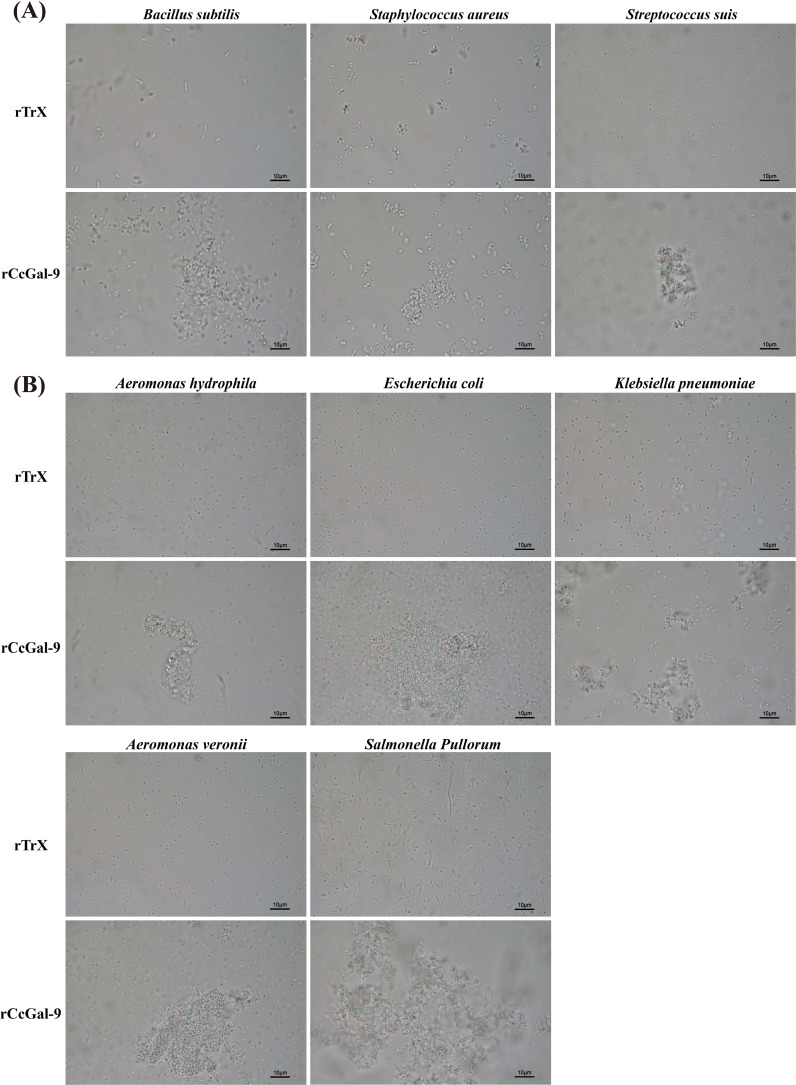
Microbial agglutination activity of rCcGal-9. **(A)** The agglutination of Gram-positive bacteria. **(B)** The agglutination of Gram-negative bacteria. Agglutinating activity were observed under a light microscope (10 × 100).

The agglutination activity of rCcGal-9 against various bacterial strains was evaluated by co-incubation using the oil immersion method. The tested strains included both Gram-positive (*M. lysodeikticus*, *B. subtilis*, *S. aureus* and *S. suis*) and Gram-negative bacteria (*A. hydrophila*, *A. veronii*, *E. coli*, *K. pneumoniae*, *P. aeruginosa*, *V. fluvialis* and *S. Pullorum*). The results demonstrated that rCcGal-9 effectively agglutinated all tested bacterial strains, with the exception of *M. lysodeikticus*, *P. aeruginosa* and *V. fluvialis*. Additionally, no agglutination was observed in the rTrx control group (**Figure 6**). The minimum agglutination concentration of rCcGal-9 was summarized in **Table 3**.

**Table 3:** Minimum agglutinating concentration of rCcGal-9 against bacteria.

Microorganisms	Minimum agglutinating concentration (µg/mL)
	rCcGal-9
Gram-positive bacteria	
*B. subtilis*	3.125
*S. aureus*	6.25
*S. suis*	6.25
Gram-negative bacteria	
*A. hydrophila*	3.125
*E. coli*	12.5
*K. pneumoniae*	12.5
*A. veronii*	6.25
S. *Pullorum*	3.125

### 3.6 Bacterial binding activity of rCcGal-9

**Figure 7:**
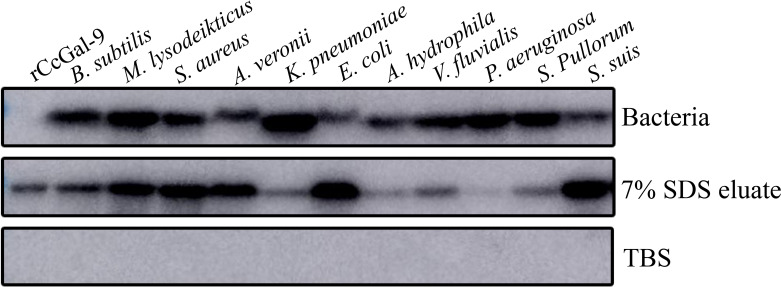
Bacterial binding activity of rCcGal-9. Bacteria as the bacterial pellet, 7% SDS eluate as the supernatant after 7% SDS elution, and TBS as the final wash solution. rCcGal-9 was incubated with bacteria as positive control.

To investigate whether rCcGal-9 could directly bind to pathogens, binding activity assays were performed. Direct binding assays were conducted to evaluate the ability of rCcGal-9 to interact with both Gram-positive and Gram-negative bacterial strains. Western blot analysis was performed, with TBS used as the final washing solution. The 7% SDS eluate represented the supernatant collected after elution with 7% SDS, while the bacterial precipitates were referred to as “bacteria”. The detection of recombinant protein in the bacterial precipitate indicated strong bacterial binding, whereas weak bands observed in the 7% SDS eluate suggested limited binding activity. The results demonstrated that rCcGal-9 bound to all tested microorganisms. Notably, rCcGal-9 exhibited weak binding activity towards *A. veronii*, *E. coli*, *A. hydrophila* and *S. suis*, whereas it showed strong affinity for the other seven bacterial strains (**Figure 7**).

### 3.7 Identification of rCcGal-9 binding to carbohydrates

**Figure 8:**
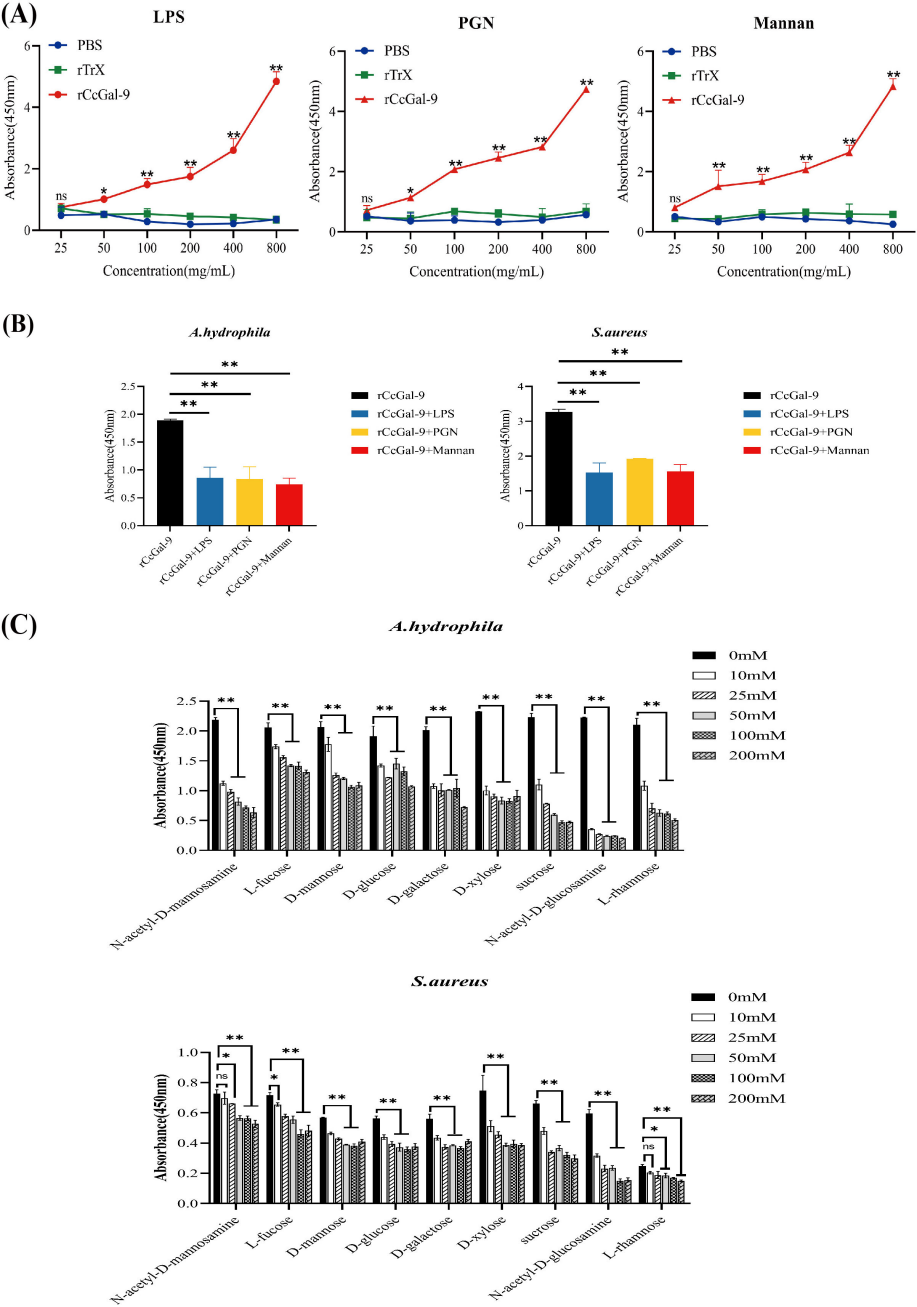
Detection of rCcGal-9 binding to bacteria and carbohydrates. **(A)** Binding of polysaccharides (LPS, PGN and mannan) to rCcGal-9 at varying concentrations was detected by ELISA. **(B)** Effects of different types of polysaccharides on rCcGal-9 binding to microorganisms. **(C)** The effects of different types of sugars on rCcGal-9 binding to microorganisms were analyzed by ELISA. The x-axis in **(C)** represents different types of sugars. Error bars represent SD (n=3), and a significant difference is indicated by asterisks (*P < 0.05, **P < 0.01).

Bacterial aggregation and binding assays revealed that rCcGal-9 is capable of agglutinating and binding both Gram-positive and Gram-negative bacteria. To further elucidate the specific pathogen-associated molecular patterns (PAMPs) involved, an ELISA was performed to assess the interaction between rCcGal-9 and various PAMPs. As shown in **Figure 8A**, rCcGal-9 exhibits a dose-dependent binding activity towards PAMPs present on bacterial cell walls, including LPS, PGN and mannan, suggesting that rCcGal-9 can bind to these components. Furthermore, competitive ELISA analysis revealed that the binding activity of rCcGal-9 to bacteria is significantly affected by the presence of LPS, PGN and mannan. Specifically, the presence of these PAMPs significantly reduced the binding of rCcGal-9 to *S. aureus* and *A. hydrophila* compared to the control groups (**Figure 8B**). Additional competitive ELISA assays demonstrated that specific concentrations of N-acetyl-D-mannosamine, L-fucose, D-mannose, D-glucose, D-galactose, D-xylose, sucrose, N-acetyl-D-glucosamine and L-rhamnose were capable of inhibiting both rCcGal-9 and bacterial binding activity (**Figure 8C**). Notably, N-acetyl-D-glucosamine and L-rhamnose were identified as significant ligands of rCcGal-9, as demonstrated by their pronounced inhibitory effects on its binding activity. These findings validate the biological activity of the purified rCcGal-9 and highlight its pivotal role in pattern recognition.

### 3.8 *In vivo* administration of rCcGal-9 promotes the defense of *A.hydrophila* infection

**Figure 9:**
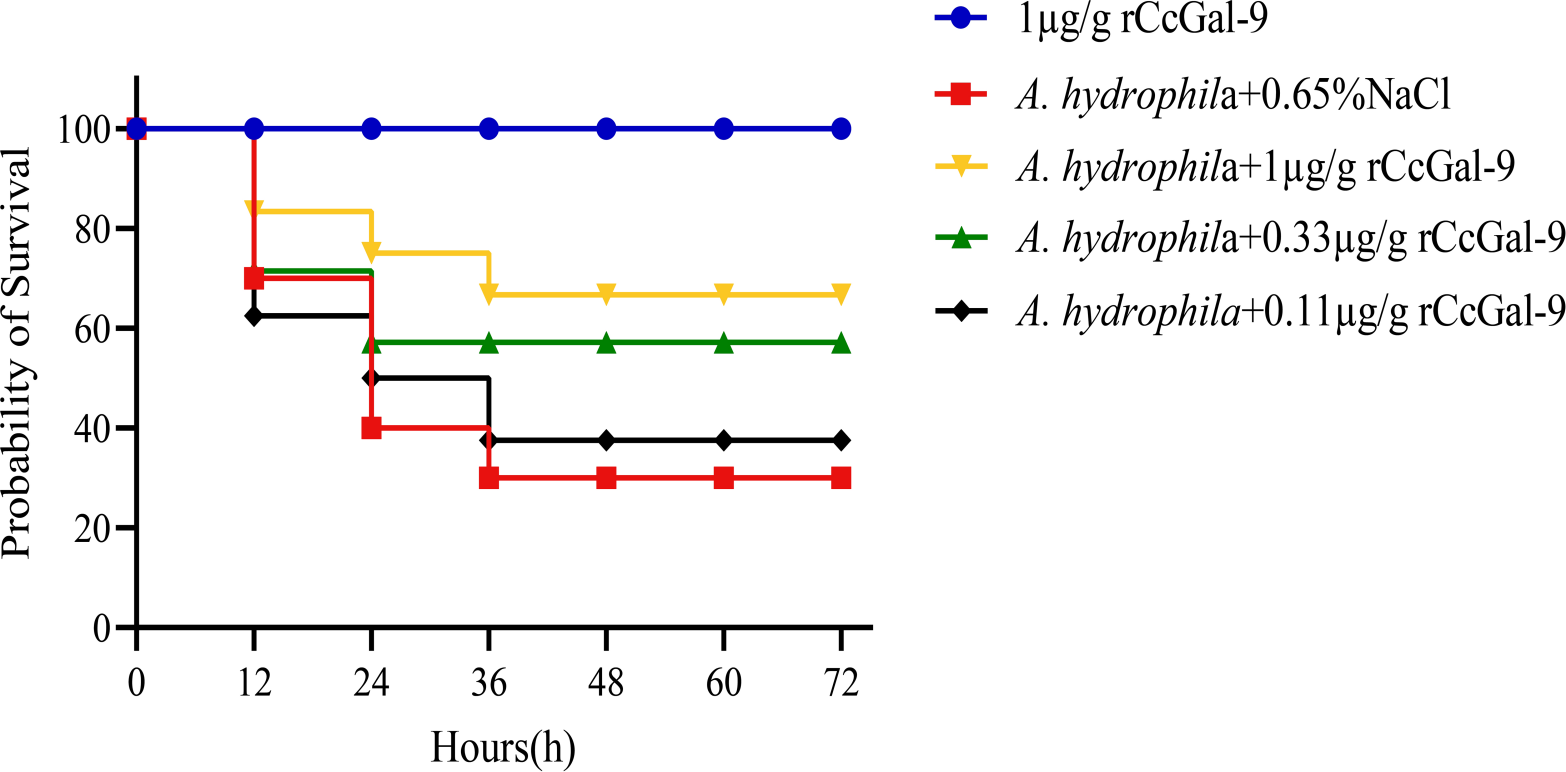
Effect of rCcGal-9 on the survival probability of *A. hydrophila* infected *C. carpio* (10 fish for each group). Group survival rates for each treatment were analyzed by the log-rank test in GraphPad Prism9 software. *A.hydrophila*+0.65%NaCl *VS* 1μg/g rCcGal-9: P=0.0172; *A.hydrophila*+0.65%NaCl *VS A.hydrophila*+1μg/g rCcGal-9: P=0.0953; *A.hydrophila*+0.65%NaCl *VS A.hydrophila*+0.33μg/g rCcGal-9: P=0.3696; A.hydrophila+0.65%NaCl *VS A.hydrophila*+0.11μg/g rCcGal-9: P=0.8214.

To assess the antibacterial function of rCcGal-9, different concentrations of rCcGal-9 were administered to *C. carpio* infected with *A. hydrophila*, and the fish’s recovery was subsequently monitored. As shown in **Figure 9**, supplementation with different concentrations of rCcGal-9 led to varying degrees of improvement in the survival rate of *C. carpio* infected with *A. hydrophila*, with the most pronounced effect observed at a concentration of 1 µg/g. These results further support the protective and regulatory role of rCcGal-9 in host defense against bacterial infections.

## 4 Discussion

Galectin-9 is an evolutionarily conserved member of the galectin family, characterized by two distinct CRDs, each containing conserved motifs (HxNPR and WGxEE) (34, 35). These conserved CRDs are essential for the carbohydrate-binding activity of galectin-9 (22, 36, 37). The HxNPR and WGxEE motifs play a crucial role in β-galactoside binding and are highly conserved among galectin-9 proteins across different species (38). In this study, a tandem repeat form of galectin-9, designated *CcGal-9*, was identified in *C. carpio*. The derived amino acid sequence of *CcGal-9* encompasses all the key characteristics of tandem repeat galectins, with its 320 amino acids containing two distinct CRDs. Notably, the WG-EER sequence at the C-terminal end has been modified to WG-EEC, suggesting that CcGal-9 may exhibit conserved binding characteristics similar to its homologs, while potentially possessing unique biological functions that warrant further investigation. Studies on teleost galectin-9 have indicated that CaGal-9 lacks both signal peptides and transmembrane domains (39). The amino acid sequence of CcGal-9 shows a similarity ranging from 31.68% to 88.44% with that of other invertebrate galectin-9 proteins. Phylogenetic analysis further demonstrates that CcGal-9 clusters with other teleost galectin-9 proteins, reinforcing its classification within conventional taxonomic frameworks.

In teleosts, galectin-9 transcripts are widely distributed across various tissues, with expression patterns differing among species (21, 22, 39–45). In the present study, *CcGal-9* was found to be broadly expressed in all examined tissues, highlighting its versatility, which is consistent with previous findings (46, 47). Notably, *CcGal-9* mRNA exhibited high expression levels in the spleen, testicle, head kidney and kidney, while moderate expression was observed in the skin, gill, brain, muscle, ovary and heart. The pronounced expression in the spleen may be attributed to the high density of immune cells, including B cells, T cells and macrophages, underscoring its role in cellular immunity. This abundant expression in immune-related organs is consistent with the galectin-9 levels observed in *Korean rose bitterling* and *Japanese flounder* (37, 41). The elevated expression of *CcGal-9* in immune tissues such as the spleen, head kidney and kidney, as well as in reproductive organs like the testicle and ovary, suggests a specialized or unique role for *CcGal-9* in the immunity of *C. carpio*.

To further investigate the immune function of *CcGal-9*, several immune-related tissues of *C. carpio*—including the liver, spleen, kidney, head kidney, intestine and gill—were selected for analysis. The expression levels of *CcGal-9* were then assessed following exposure to *A. hydrophila* and *S. aureus*. The results revealed a significant upregulation of *CcGal-9* in *A. hydrophila*-infected *C. carpio*, which is consistent with previous findings regarding *TfGal-9* in *T. fasciatus* infected with LPS (16). Unexpectedly, *CcGal-9* expression was downregulated in both the kidney and gill tissues following stimulation with *A. hydrophila* and *S. aureus*, respectively. This downregulation parallels the response of human macrophage-derived galectin-9 to *M. tuberculosis* infection, as well as the expression pattern of *LcGal-9* in *L. crocea* in response to Poly I:C and *Vibrio stimulation* (40, 48). Variations in the results may be attributed to two primary factors. First, differences in bacterial dosage and host genetic background can influence immune responses, as pathogens employ diverse strategies to evade host defenses. Another potential factor is the immune evasion strategies employed by *A. hydrophila*, as supported by evidence indicating that *V. parahaemolyticus* adapts to the hostile intracellular environment of macrophages (49). Following administration of *S. aureus* to the fish, *CcGal-9* expression levels were assessed in various tissues, including the liver (12 to 72 hpi), spleen (3 to 72 hpi), kidney (3 and 72 hpi), head kidney (3 to 72 hpi), intestine (6 to 72 hpi) and gill (24 to 48 hpi). This significant upregulation suggests the occurrence of a bacterial-induced inflammatory response during the post-infection period. These results indicate that *CcGal-9*, functioning as a PRR, plays a crucial role in the immune response to bacterial infections.

Galectins exhibit agglutination activity, a hallmark feature that enables them to aggregate viruses, bacteria, protozoa and fungi through binding to glycans present on their surfaces (37). In teleosts, recombinant galectin-9 has demonstrated the ability to aggregate a broad spectrum of microorganisms, including Gram-positive and Gram-negative bacteria, as well as fungi (41, 42, 50). In *C. carpio*, rCcGal-9 exhibited agglutination activity against all tested microorganisms, including both Gram-positive and Gram-negative bacteria. This finding indicates that rCcGal-9 possesses broad aggregation capabilities against a wide range of detected microorganisms. This observation was consistent with previous studies, in which rCaGal-9 from *C. auratus* was shown to aggregate all identified bacterial strains, including three Gram-positive strains (*B. subtilis*, *S. aureus*, and *S. suis*) and five Gram-negative strains (*A. hydrophila*, *E. coli*, *K. pneumoniae*, *A. veronii* and *S. Pullorum*) (42).

The agglutination activity of rCcGal-9 is mediated by its direct interaction with microorganisms. To elucidate the underlying mechanism, we evaluated the binding activity of rCcGal-9 toward various microbial species. These findings are consistent with previous studies (43), for instance, rPfGAL9 exhibits selective binding to microorganisms. In contrast, our results demonstrate that rCcGal-9 is capable of binding to all tested microbial species, albeit with differing binding activities. The observed variation in binding correlates with the size of aggregates formed by agglutinated bacteria, suggesting that the broad-spectrum affinity and specificity of rCcGal-9 may be attributed to its highly conserved sugar-binding sites and the presence of two structurally distinct CRDs. Binding and agglutination assays demonstrated that rCcGal-9 is capable of binding to *P. aeruginosa* and *V. fluvialis*, although it does not induce agglutination. This observation is consistent with findings for FcLec4 from *Chinese white shrimp* (51) and TfCTL1 from *T. fasciatus* (52). We hypothesize that the weak binding between the lectin and these bacteria may be insufficient to induce aggregation. Consequently, further investigations are necessary to elucidate the detailed mechanisms underlying the interaction between rCcGal-9 and bacteria.

Galectin-9 is known to recognize exogenous glycans on the surface of microorganisms, facilitating their aggregation by binding to individual glycans or glycan complexes, thereby promoting microbial clearance (37, 53). This protein plays a critical role in pattern recognition by interacting with a broad spectrum of microbial surface carbohydrates, a process essential for the host’s innate immune response. In the present study, we demonstrate that rCcGal-9 can bind to LPS, PGN and mannan, findings that are consistent with previous reports in *C. auratus* and *Crassostrea gigas* (54). Our results further reveal that rCcGal-9 exhibits broad binding specificity, interacting with nine distinct saccharides, with a notably higher affinity for L-fucose and D-mannose. These observations are consistent with the reported functionalities of mammalian galectin-9, suggesting evolutionary conservation of carbohydrate recognition across phylogenetically distant species (55, 56). Lectins serve as crucial components of PRRs, recognizing specific carbohydrate moieties on the surfaces of pathogenic cells. Through this recognition, lectins promote pathogen agglutination and enhance their susceptibility to phagocytosis, thereby playing a key role in the host’s innate immune defense (57). Moreover, several lectins have been shown to exhibit potent antimicrobial activity by binding specific carbohydrate structures on microbial surfaces (35, 58). In the present study, we observed that elevated concentrations of rCcGal-9 exerted significant antibacterial effects against *S. aureus* and *A. hydrophila in vitro*, consistent with findings previously reported in *Nile tilapia* (34). The antibacterial mechanism of CcGal-9 appears to resemble that of certain antimicrobial peptides, involving interactions with bacterial cell wall components, which disrupt membrane integrity, promote pore formation, and lead to the leakage of intracellular contents (59, 60).

*In vivo* experiments revealed that supplementation with rCcGal-9 significantly enhanced the survival rate of *C. carpio* following *A. hydrophila* infection. This protective effect is presumably mediated by the potent immune response induced upon rCcGal-9 administration.

In conclusion, a tandem-repeat type galectin-9, named *CcGal-9*, was identified in *C. carpio*. CcGal-9 contains two distinct CRDs and is widely expressed across various tissues, with the highest expression observed in the spleen. Following challenge with *S. aureus* and *A. hydrophila*, the transcriptional levels of *CcGal-9* exhibited significant fluctuations in several immune-related organs, including the liver, spleen, kidney, head kidney, intestine and gill. These findings suggest that *CcGal-9* plays a pivotal role in the innate immune response of *C. carpio* against bacterial infections. The rCcGal-9 exhibits potent microbial agglutination activity, effectively agglutinating eight distinct bacterial strains. Furthermore, rCcGal-9 was shown to bind a broad range of PAMPs as well as all tested microbial species. Collectively, these results suggest that rCcGal-9 functions as a key PRR in the innate immune defense of *C. carpio*, mediating the recognition of PAMPs on the surfaces of pathogenic microorganisms. This study provides valuable insights into the immunological role of galectin-9 in teleost fish, underscoring its significance in host-pathogen interactions.

## Data Availability

The datasets presented in this study can be found in online repositories. The names of the repository/repositories and accession number(s) can be found in the article/supplementary material.
